# In situ characterization of hydrogen absorption in nanoporous palladium produced by dealloying

**DOI:** 10.3762/bjnano.7.110

**Published:** 2016-08-17

**Authors:** Eva-Maria Steyskal, Christopher Wiednig, Norbert Enzinger, Roland Würschum

**Affiliations:** 1 Institute of Materials Physics, Graz University of Technology, Petersgasse 16, A-8010 Graz, Austria; 2 Institute of Materials Science and Welding, Graz University of Technology, Kopernikusgasse 24, A-8010 Graz, Austria

**Keywords:** dealloying, dilatometry, hydrogen storage, nanoporous palladium, resistometry

## Abstract

Palladium is a frequently used model system for hydrogen storage. During the past few decades, particular interest was placed on the superior H-absorption properties of nanostructured Pd systems. In the present study nanoporous palladium (np-Pd) is produced by electrochemical dealloying, an electrochemical etching process that removes the less noble component from a master alloy. The volume and electrical resistance of np-Pd are investigated in situ upon electrochemical hydrogen loading and unloading. These properties clearly vary upon hydrogen ad- and absorption. During cyclic voltammetry in the hydrogen regime the electrical resistance changes reversibly by almost 10% upon absorbing approximately 5% H/Pd (atomic ratio). By suitable loading procedures, hydrogen concentrations up to almost 60% H/Pd were obtained, along with a sample thickness increase of about 5%. The observed reversible actuation clearly exceeds the values found in the literature, which is most likely due to the unique structure of np-Pd with an extraordinarily high surface-to-volume ratio.

## Findings

The knowledge about the remarkable hydrogen absorption properties of palladium dates back to the mid-nineteenth century [1] and has been studied intensively ever since. Even though palladium itself does not possess technical relevance as a hydrogen storage material due to its high price and rather low achievable H storage densities, it is a frequently used model system. During the past few decades particular interest was placed on nanostructured Pd, which exhibits superior absorption properties due to a high surface-to-volume ratio, and its application potential in important fields of technology such as energy storage, sensing and catalysis [2].

One attractive method to produce nanostructured metals with macroscopic dimensions is dealloying, an (electro-)chemical process, which removes the less noble component from an alloy by selective etching [3]. Nanoporous palladium (np-Pd) produced by free corrosion [4] as well as potential-assisted dealloying [5] has been studied recently with regards to actuation upon electrochemical hydrogenation [4] as well as hydrogen solubility from the gas phase [6]. In the present study np-Pd is produced by electrochemically dealloying a Co–Pd master alloy and investigated upon electrochemical H absorption in an alkaline aqueous electrolyte. Volume and electrical resistance of the sample, two qualities both known to sensitively depend on the H content of Pd [7–8,4,9–11], are monitored in situ during electrochemical hydrogenation and compared to literature data.

The Co–Pd master alloy (80 wt % Co–20 wt % Pd) was prepared by electron beam melting (EBM) high purity wires of Co and Pd (ChemPur, 99.99%). The resulting alloy droplet was rolled to a thickness of 280 μm in several steps. Between each rolling step, the alloy was annealed for 1 h at 800 °C. From this master alloy sample platelets were cut for dealloying and subsequent charging experiments.

The resistometric measurements were set up similar to our work on nanoporous platinum [12–13]. To sum up briefly, the rectangular sample (ca. 12 × 3 mm^2^) was immersed into the electrolyte hanging on five Pd wires, glued onto the platelet. The mid-positioned wire served as working-electrode contact connected to a PGZ-100 potentiostat (Radiometer Analytical), the other wires were connected to a Keithley 2400 Source Meter for four-point resistometry.

For dilatometry a square shaped sample platelet (ca. 5 × 5 mm^2^) was placed under the pushrod of a Linseis L75 vertical dilatometer applying a constant pressure of 100 mN. A well-annealed, flattened Pd wire served as working electrode contact to an Autolab PGSTAT204 potentiostat.

Electrochemical charging was performed at room temperature using commercial Ag/AgCl (saturated KCl) reference electrodes, related to which all potentials *U*_Ag/AgCl_ will be stated. Co–Pd was dealloyed in 0.1 M H_2_SO_4_ under the same conditions as in [5], which were shown to yield porous structures with pore diameters of 5–20 nm [5] and very low residual Co concentrations [6]. Correspondingly hydrogen concentrations were calculated assuming Co-free samples in the following. With this assumption a specific surface area of about 30 m^2^/g was determined electrochemically for np-Pd from monolayer adsorption of oxygen, following a procedure presented in detail in reference [14]. All electrochemical experiments subsequent to dealloying were performed in a 1 M solution of KOH, a highly porous carbon fabric contacted by a Pd wire served as counter electrode. The property variations of np-Pd upon electrochemical charging were calculated with respect to the sample resistance *R*_0_ and thickness *L*_0_ in the electrochemical double layer regime.

**Figure 1:**
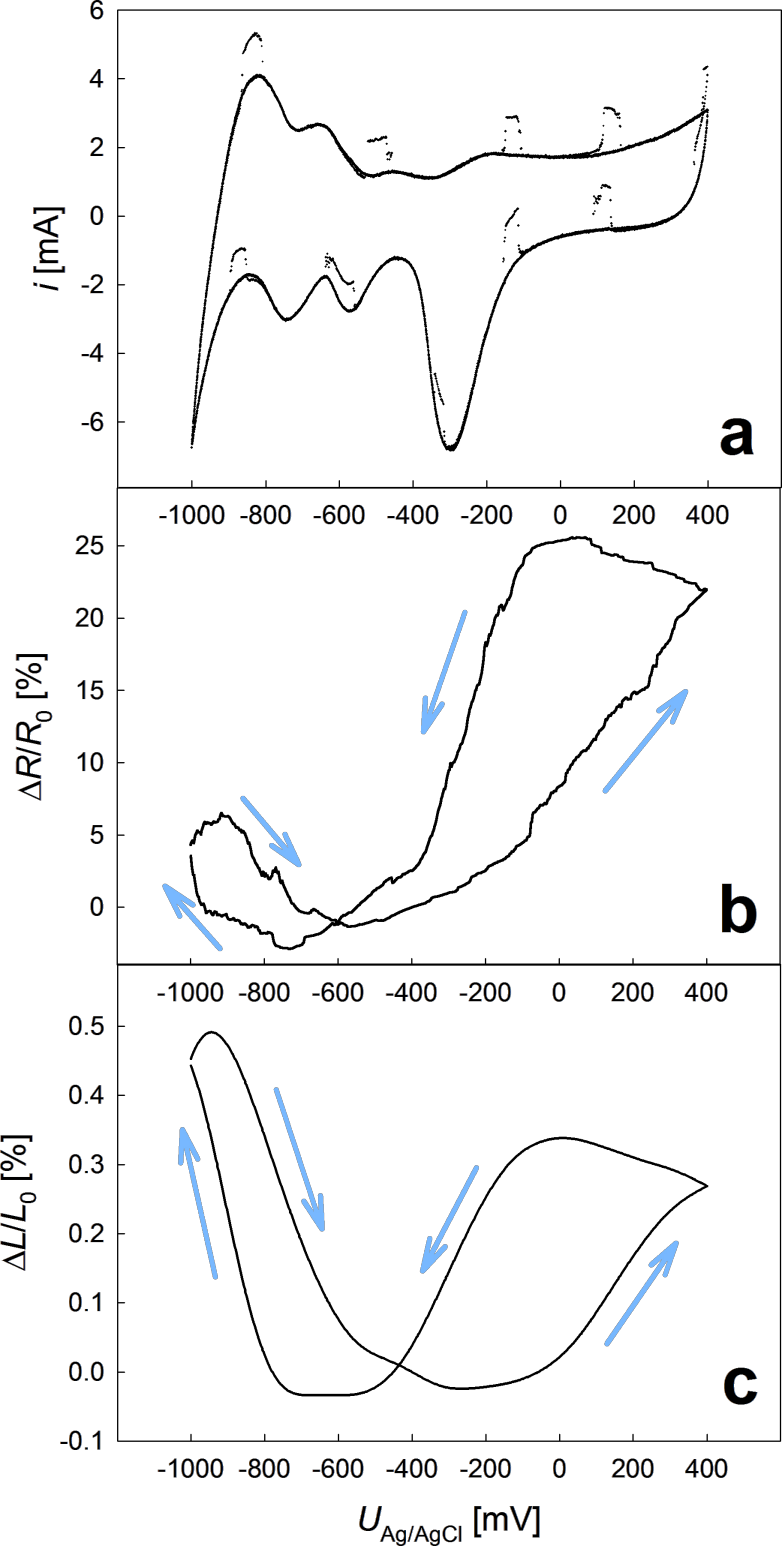
Cyclic voltammetry of np-Pd with a scan rate of 1 mV/s, recorded between potentials *U*_Ag/AgCl_ of −1000 mV and +400 mV (a) with concommitant relative variations in electrical resistance (b) and sample thickness (c).

Nanoporous metals freshly prepared by dealloying are in a strongly oxidized condition, associated with particular physical properties [15–16,12–13]. By suitable treatment, i.e., repeated cyclic voltammetry (CV) in a wide potential range, the sample can be reduced until finally a metallic CV behavior is reached in a steady state. Figure 1a shows a steady-state CV performed with a scan rate of 1 mV/s between potentials *U*_Ag/AgCl_ of −1000 mV and +400 mV. As the potential varies triangularly, the CV curve is run through clockwise, while resistance changes up to almost 30% (Figure 1b) and thickness variations of about 0.5% (Figure 1c) occur. Occasionally visible spikes in the otherwise smooth cyclic voltammogram are caused by coinciding resistometric measuring pulses, which have to be sufficiently high for accurate resistance results. This causes certain irregularities in the CV curve, yet does not interfere with the property-tuning experiment.

In the double layer and oxygen regime (potentials between −550 mV and +400 mV) the property variations are similar to previous observations for porous nanophase metals: Increasing sample thicknesses and resistances upon positive scan are considered to be caused by charging-induced modifications in the atomic bindings [17–20] and charge-carrier scattering [16,21,13] at the metal surface, respectively. The hydrogen regime, situated at potentials between −1000 mV and −550 mV, which is particularly interesting due to the extraordinary H-absorption properties of Pd, is associated with resistance variations Δ*R*/*R*_0_ of approximately 7% and a thickness increase Δ*L*/*L*_0_ of about 0.5%.

**Figure 2:**
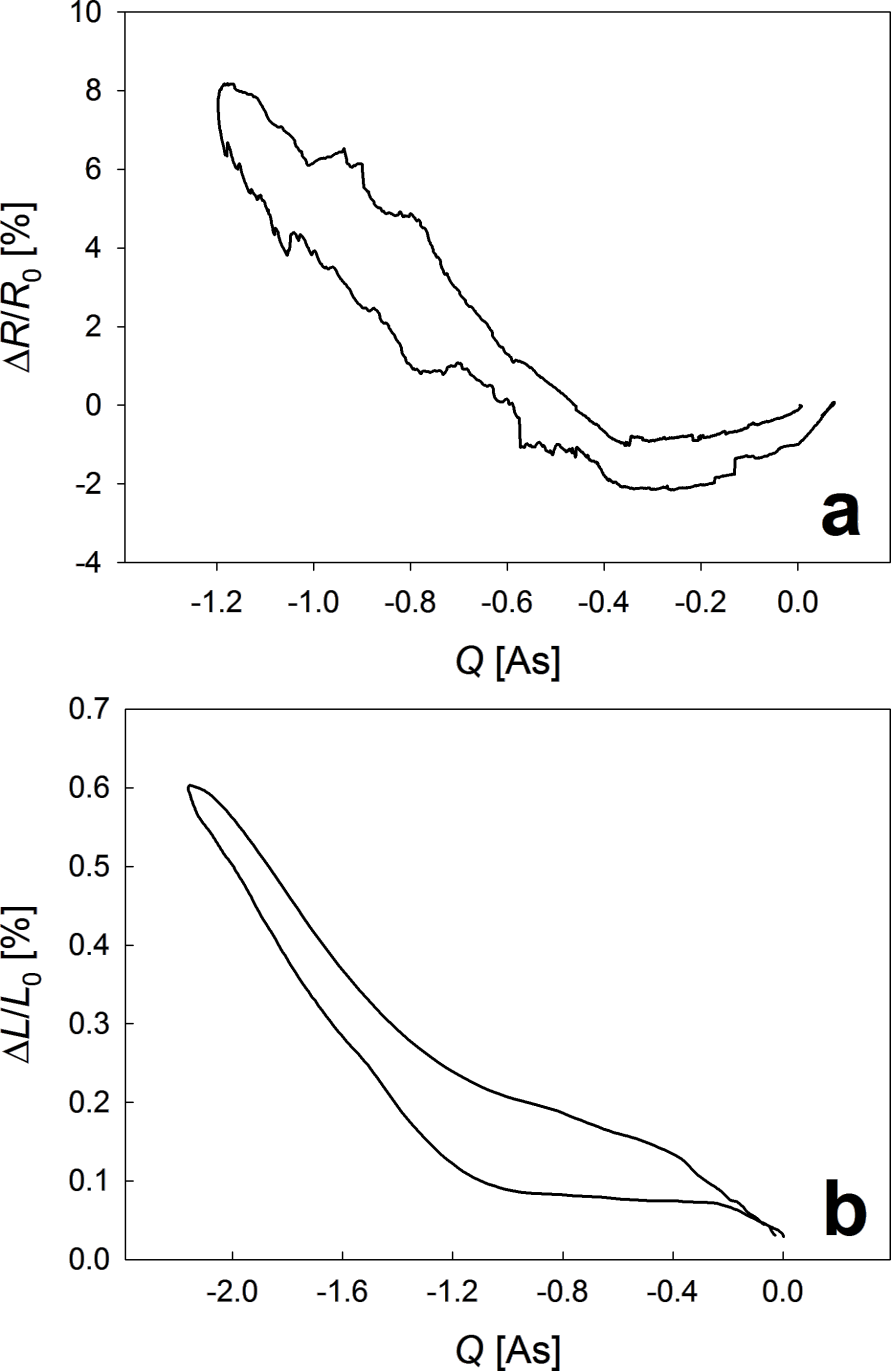
Charge dependence of relative electrical resistance (a) and sample thickness (b) upon cyclic voltammetry in the hydrogen regime at potentials *U*_Ag/AgCl_ between −1000 mV and −550 mV.

This regime was investigated in more detail by cyclic voltammetry at a very slow scan rate of 0.1 mV/s. Figure 2 shows the relative variations of electrical resistance and sample thickness as functions of the imposed charge *Q*, determined by continuously integrating the charging current, corrected for minor leakages by subtracting a constant factor determined from the cycle to cycle offset. Resistance and length changes reveal remarkable similarities, both exhibiting two regions of different slope, attributed to hydrogen adsorption followed by hydrogen absorption at higher imposed charges. For dilatometry (Figure 2b) these results agree fairly well with the behavior of consolidated palladium nanoparticles, which was discussed within an electrocapillary coupling model by Viswanath and co-workers [8].

For the resistance variation (Figure 2a), the total charge flow of about −1.2 A·s arises from a transfer of about −0.3 A·s in the adsorption and −0.9 A·s in the absorption regime. Converting the latter into the absorbed hydrogen concentration *c* yields a H/Pd atomic ratio of about 5%, associated with a variation of *R* of about 9.6%. At such low hydrogen concentrations, the electrical resistance of bulk Pd is particularly sensitive to hydrogen absorption. Values for the resistance coefficient (Δ*R*/*R*_0_)/*c* deduced from literature data range from 1.8 [9] to 2.3 [10].

The matching of these values with the present resistance coefficient (Δ*R*/*R*_0_)/*c*=1.9 of np-Pd is, however, surprising: Since nanoporous metals possess a high initial resistance, governed by surface scattering processes [16], a lower sensitivity to additional scattering centers inside the crystal due to absorbed hydrogen would be expected. An additional resistance contribution by adsorbates might arise in the present case, in analogy to the oxygen regime of porous nanophase metals [16,21].This is, however, unlikely as the variations of *R* during adsorption are small. The striking similarities between Δ*R*/*R*_0_ and Δ*L*/*L*_0_ suggest that actuation might influence *R*: An expansion during hydrogenation may disconnect electrical contacts in the nanoporous network, leading to a resistance increase. Such a mechanical effect would also explain the noisy *R*-signal due to immediate resistance changes caused by opening/closing connections.

**Figure 3:**
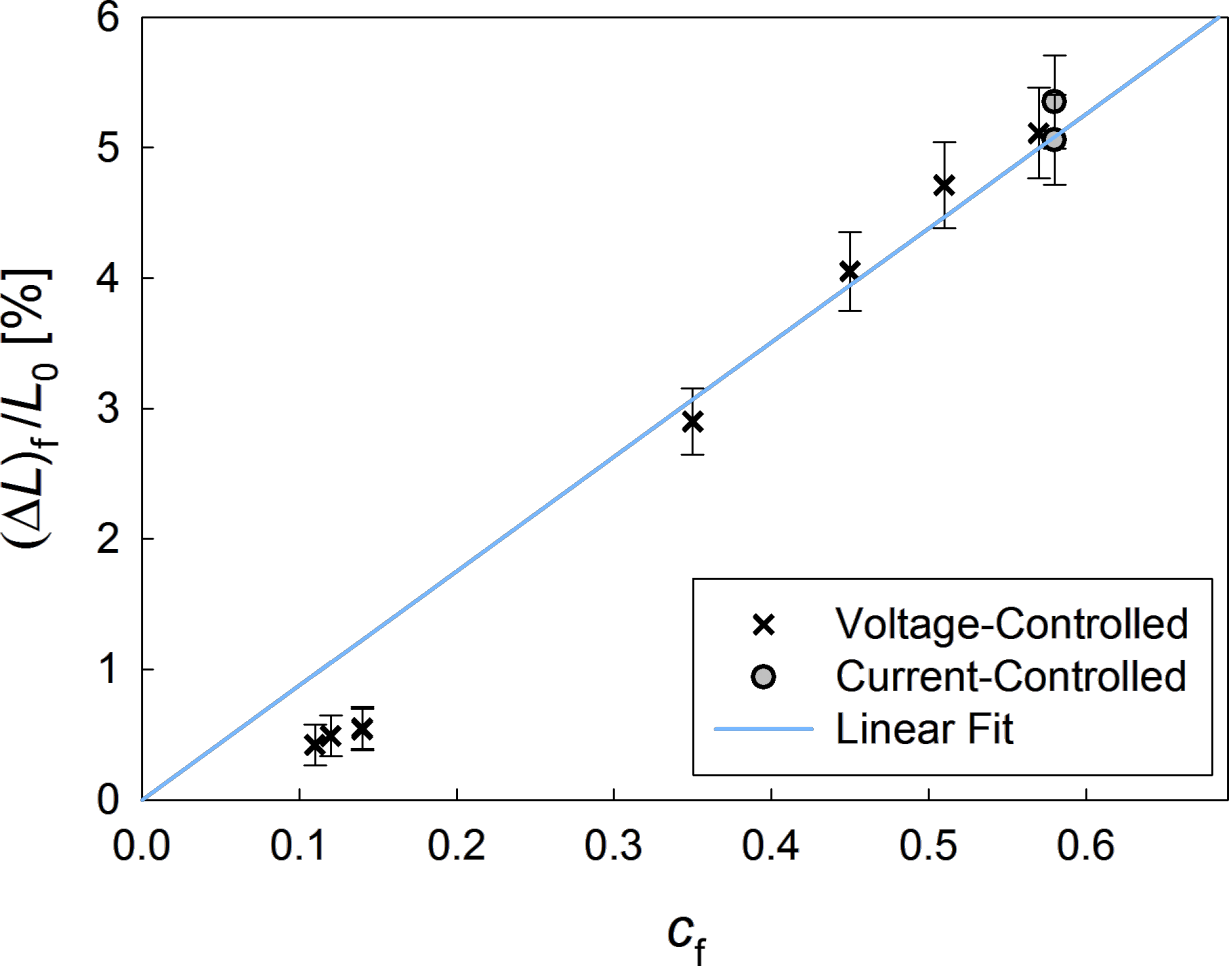
Thickness variations (Δ*L*)_f_/*L*_0_ of np-Pd upon loading experiments up to different final hydrogen concentrations characterized by the H/Pd atomic ratio *c*_f_ (see Table 1).

Since extraordinarily strong actuation properties were reported quite recently for nanoporous Pd produced by free corrosion [4], the dilatometric measurements were extended to higher hydrogen concentrations by employing different charging parameters (Table 1). Before each loading experiment, the sample was activated by a CV similar to Figure 1. Hydrogen was absorbed either voltage-controlled at constant potentials between −900 mV and −1050 mV, or current-controlled by applying a constant current and in both cases subsequently desorbed at a constant potential of −400 mV for 120 min. The stored amount of hydrogen was determined by integrating the discharging current. The length changes measured along with the hydrogen charging are plotted in Figure 3.

**Table 1:** Dilatometrically monitoring hydrogen absorption upon voltage-controlled charging procedures at quoted voltages *U*_Ag/AgCl_ or current-controlled charging at quoted currents, resulting in a final H/Pd atomic ratio *c*_f_ associated with a final thickness increase (Δ*L*)_f_/*L*_0_ (see Figure 3).

parameters	time [min]	*c*_f_	(Δ*L*)_f_/*L*_0_ [%]

−900 mV	60 120	0.12 0.11	0.49 0.42
−950 mV	60 120	0.14 0.14	0.54 0.55
−1000 mV	60 120	0.35 0.45	2.90 4.05
−1050 mV	60 120	0.51 0.57	4.71 5.11
−5 mA	60	0.58	5.35
−2 mA	150	0.58	5.06

Upon charging to a H/Pd ratio of almost 60%, a thickness increase of about 5% was observed, representing the highest electrochemically induced actuation reported for a dealloyed material so far. This variation is not only significantly higher than predicted by literature data for bulk Pd [7], according to which an approximately linear variation of only about 3.5% would be expected at this concentration, but also exceeds the maximum hydrogen solubility as well as the relative sensitivity of actuator response to hydrogen loading of recently investigated nanoporous palladium produced by free corrosion of an Al–Pd master alloy [4].

While the maximum hydrogen solubilities show a strong variation with the charging parameters, such as the chosen electrolyte and applied potential, the strong sample expansion upon hydrogenation is directly related to the nanoporosity of np-Pd, i.e., its high surface-to-volume ratio. Since nanoporous Pd possesses very fine pore sizes of 5–20 nm [5,22–23], which strongly depend on the dealloying parameters, a significant fraction of the total H absorption takes place on sites close to the surface. Being subjected to significantly lower constraints than atomic layers inside the bulk, superficial planes may show stronger outward relaxation during hydrogen uptake, similar to the surface-chemistry-driven actuation behavior known for nanoporous gold, which is caused by alterations in the atomic bindings in superficial atomic layers [19].

In conclusion, resistometry and dilatometry represent two efficient, independent methods to characterize the hydrogen concentration in nanoporous palladium. The volume expansion of nanoporous palladium upon hydrogen absorption is stronger than predicted by literature data, which might be due to the high surface-to-volume ratio in the nanoporous samples. Resistance variations of almost 10% could be achieved upon absorbing approximately 5% H/Pd during cyclic voltammetry. Besides scattering processes also a resistance contribution by actuation in the nanostructure was discussed. To give a brief outlook, the H absorption behavior of np-Pd should be studied systematically with regards to different master alloy compositions and dealloying parameters. Moreover the experiments might be extended to higher hydrogen concentrations by using acidic electrolytes in future experiments.
